# The Defective and Dependent


**Published:** 1912-01-06

**Authors:** 


### THE DEFECTIVE AND DEPENDENT.*

### IV. Education of the Mentally Defective.

The axiom on which all successful training of mentally defective children is based was long ago enunciated by Séguin in the words, "The physiological training of the senses must precede the psychical education of the mind." This principle indeed applies to all children, but it is of special importance in the case of those whose senses are functionally inactive, if not structurally incomplete, and whose powers of observation lag behind instead of advancing with age by a species of natural evolution. What comes spontaneously to the normal child has, indeed, to be made a matter of specific instruction in the case of the abnormal; the senses of touch, sight, hearing have all to be stimulated and disciplined, and, what is most essential of all, the faculty of attention strengthened so that sensations may be at last registered in the brain as perceptions and stored there for comparison in the formation of ideas. Furthermore, the infirmity of will and imperfect powers of co-ordination characterising the mentally defective child have to be reckoned with, and associated physical disabilities as far as possible remedied. Thus the problem of the education of the mentally defective child concerns not only the teacher but the psychologist and physician, and the system evolved has been aptly described as "medico-pedagogic."

In the last paper we saw that in this country two classes of agencies have taken part in the training of the mentally defective: (1) the asylums for imbeciles, which have dealt with all classes of mental defect; and (2) the special schools established under the Act of 1899 to meet the needs of children found incapable of benefiting by the ordinary curriculum of the elementary schools. We propose now to describe in some detail the work of the latter and to point out the desiderata which would render them more serviceable to the community.

In the first place we must draw attention to the fact that the Act of 1899 being permissive only and not compulsory, no provision exists in 180 educational districts (out of 322 in England and Wales) for the special training of mentally defective children, and as a matter of fact only about 40 of the more important authorities have established special schools of their own, to which, however, in many cases other authorities contribute and send children. The Chief Medical Officer of the Board of Education estimates in his last annual report that not more than one-fourth of the necessary provision has been so far brought into existence, the 162 certified day schools and seven residential schools only affording accommodation for about 12,000 out of the 48,000 mentally defective children of school age requiring special instruction.

The Act of 1899 gives power to a local education authority to ascertain what children in its area are defective (mentally or physically), and wherever such investigations have been held the result tends to confirm the statistics of the medical investigators to the Royal Commission that on an average about 0.79 per cent. of the number of children on the school registers will prove to be mentally defective. Though the percentage appears to vary considerably in different localities, being highest in urban areas, there is ample evidence to justify the compulsory application of the Act throughout the country, which would include the provision of boarding schools for those belonging to rural districts with scattered populations.

Children are admitted to the special school on the certificate of a duly qualified medical officer (approved by the Board of Education) to the effect that, though not imbecile or merely dull and backward, they are capable of receiving benefit from instruction in the special school though incapable, by reason of mental or physical defect, from receiving proper benefit at the ordinary school. The reasons for forming this opinion are not required in the certificate, but are usually recorded in the register of progress kept at the school, and the condition of the pupil is each year ascertained by the medical officer as to whether (1) he is fit for promotion to an ordinary elementary school, or (2) he is ineducable even in the special school.

The curriculum of the special school differs from that of the ordinary school in the following points: (1) The more objective character of the instruction individualised as much as the comparatively small size of the classes (not more than twenty pupils to one teacher) renders practicable; (2) increased attention paid to physical and specially to manual training; (3) shorter hours of attendance and shorter lessons, so as not to induce mental fatigue. The schools as originally organised were usually mixed schools under female teachers, but as attendance is compulsory up to the age of sixteen, experience has shown the desirability of setting up separate classes for the industrial training of the elder boys, and there are now in London twelve such centres under male teachers, besides one special industrial department for elder girls.

The utility of the special school system has of late years been subjected to much criticism; and in conclusion we quote some judicious remarks in the report for 1909 of the Chief Medical Officer of the Board of Education on the subject. "It must not be forgotten," he writes, "that this work is, comparatively speaking, of recent origin—indeed the first special day schools were only established in England in 1892—and the marked progress which has occurred has been accomplished under a permissive Act and in the face of exceptional difficulty. It is certainly much too early to conclude that the day special schools have failed if 40 to 45 per cent. of these afflicted children have become disciplined and employable. The history and results of these schools teach us where and how we need to concentrate in order to make them, in their right place, useful and effective. And mainly it must be said that we need better *selection, training, and after-care.*"

* Previous articles in this series have appeared in The Hospital of Nov. 4, Nov. 18, and Dec. 2.

